# Brain Encoding of Social Approach: Is it Associated With Spatial Ability?

**DOI:** 10.3389/fnbeh.2019.00179

**Published:** 2019-08-06

**Authors:** Hipólito Marrero, Elena Gámez, Mabel Urrutia, David Beltrán, Jose M. Diaz, Sara N. Yagual

**Affiliations:** ^1^Departamento de Psicología Cognitiva, Social y Organizacional, Universidad de La Laguna, San Cristóbal de La Laguna, Spain; ^2^Instituto de Neurociencias de la Universidad de La Laguna, San Cristóbal de La Laguna, Spain; ^3^Facultad de Educación, Universidad de Concepción, Concepción, Chile; ^4^Facultad de Ciencias Sociales y de la Salud, Universidad Estatal Península de Santa Elena, La Libertad, Ecuador

**Keywords:** superior temporal sulcus, social approach, spatial ability for figure mental-rotation, adaptive conduct, action understanding

## Abstract

Human brains encode approach in social relationships as cognitively relevant for adaptive behavior. In this study, using event-related potentials (ERPs), we found that reading approach-social actions are likely to cause activation around the right anterior/middle superior temporal sulcus (STS), a brain area particularly involved in processing action intentionality and social relationships. We consider that the human capacity for the mental rotation of figures could also be adaptive for social relationships at the service of planning interaction with other bodies in social encounters. Encoding of social approach and spatial ability would correlate if both capacities are aimed at achieving the adaptive goal of secure interactions with others. We found a strong correlation between brain activation in the right temporal brain region and spatial ability. Implications of these results for the psychological mechanisms involved in adaptive social behavior are discussed.

## Introduction

Approach and avoidance are basic representations of intentionality of human actions (Elliot, 2006) and also of social relationship actions (Marrero et al., 2017; see also Gámez and Marrero, 2001). Approach encodes in actions a positive attitude and closeness to, and avoidance a negative attitude and distance from, other individuals (e.g., accept/reject, include/exclude, praise/despise, help/harm, etc.). The superior temporal sulcus (STS) is part of the mentalizing network that is recruited for processing intentionality (Spunt et al., 2010; Dodell-Feder et al., 2011; Kennedy and Adolphs, 2012) and social information (see Lahnakoski et al., 2012), usually stronger in the right hemisphere (see Watson et al., 2014). In particular, the posterior STS is recruited for encoding approach intentionality in social perception (Pelphrey and Morris, 2006), whereas the anterior and middle STS are involved in processing scenes of social interactions (Iacoboni et al., 2004; Lahnakoski et al., 2012) and in judging “friendship” from social-like interactions in the Heider and Simmel animation task (Tavares et al., 2008; Ross and Olson, 2010). Notwithstanding this evidence, the role of the STS in processing approach in social relationship actions has not been directly examined in previous research. The present study explores for the first time whether approach vs. avoidance social actions involves specialized brain processing; in particular, whether the STS is recruited in their processing.

Moreover, related to approach relationships, the capacity of planning bodily interaction is necessary for individuals to move efficiently in social encounters. This planning capacity involves the mental rotation of solid figures, as rotating mentally other bodies would be an imagined action related to the planning of real actions (see Wohlschläger and Wohlschläger, 1998; see also Jolicoeur and Cavanagh, 1992). Several studies support this proposal. For example, spatial rotation tests with human figures (instead of geometrical figures) improved performance in both sexes (Amorim et al., 2006; Alexander and Evardone, 2008; Voyer and Jansen, 2016). Furthermore, it has been found that training in wrestling or juggling, but not running improves this ability (Moreau et al., 2012; see also Voyer and Jansen, 2017). We investigate whether individual differences in brain encoding for approach and spatial ability could be associated. A deeper encoding of approach would improve discrimination of safer “closed” others. Likewise, spatial ability enables more efficient bodily interactions also necessary for ensuring self-protection in social encounters. Thus, individuals who both more deeply encode relationship approach and move more efficiently in social encounters would increase their fitness and survival.

In a previous study (Marrero et al., 2017), we tested the hypothesis that understanding others’ social actions, as based on our own experience, would activate self-experienced approach/avoidance brain representations. In the study, participants’ electrophysiological activity was recorded while they were reading approach/avoidance action sentences from a character toward a target: a thing/a person (i.e., “Petra accepted/rejected Ramón in her group”/“Petra accepted/rejected the receipt of the bank”). Brain potentials time-locked to the target word were measured. We found different event-related potentials (ERPs) to things and persons, which supports specific processing for approach/avoidance actions with persons. Then, we reanalyzed the ERP data in the time window associated with persons-targets to estimate the brain sources of approach/avoidance differences. Subsequently, we correlated individual differences in activation in these brain areas with participants’ spatial ability.

## Materials and Methods

### Participants

Twenty-three (18 women) 20- to 30-year-old (mean = 22.6) healthy right-handed students at the University of La Laguna with normal or corrected to normal visual acuity participated in this study. All participants gave written informed consent in accordance with the Declaration of Helsinki. The study was approved by the Committee of Ethics of Research and of Animal Welfare of University of La Laguna (CEIBA2017-0272). The minimal sample size (Cohen, 1992; Faul et al., 2009) to generate appropriate statistical power (0.80) with 0.05 alpha bilateral for a medium to high correlation (*r* = 0.6) was calculated at 20. Twenty-two participants completed the spatial test.

### Stimuli and Procedure

The methods used in the ERP study were described previously in detail (Marrero et al., 2017). Participants were instructed to read sentences while seated in front of a computer screen. They were given 200 sentences, 40 for each experimental condition: approach-person, approach-thing, avoidance-person and avoidance-thing, and 40 filler sentences presented word by word. Each sentence was composed of nine words (for example “Petra aceptó a Ramón en su grupo de trabajo”) displayed as follows: a rate of 200 ms for articles and prepositions and 700 ms for nouns and verbs. One third of the sentences were immediately followed by a question on the content just read.

*Spatial ability to rotate solid figures test*: we applied the *Test on rotation of solid figures* (Yela, 1969). It is a 21-item psychometric paper-and-pencil test with a time limit of 6 min. Each item includes a model figure and five alternatives that must be evaluated against it. Participants must choose which alternative, rotated within a 3D space, fits the model figure.

### EEG Recording and Analysis

EEG was recorded from 60 electrodes mounted in elastic Quick-caps (Neuromedical Supplies, Compumedics Inc., Charlotte, NC, USA) arranged according to the standard 10–20 system. All EEG electrodes were referenced online to an electrode at vertex, and recomputed offline against the average reference with high- and low-pass filter set at 0.05 and 100 Hz, respectively. Independent component analysis was applied to the data to remove the effects of blinks and eye movements. Remaining trials with EEG voltages exceeding 70 μV measured from peak to peak at any channel were also removed. Baseline correction of averaged data was carried out using the time interval between 600 and 400-ms preceding the onset of the critical word.

ERP waveforms were statistically evaluated using the cluster-based random permutation method implemented in Fieldtrip (Maris and Oostenveld, 2007) applied to the 800-ms following the onset of the critical thing/person target. This method deals with multiple comparisons in space and time by identifying, over the whole ERP segment (24,000 sample points: 400 time points, and 60 channels), clusters of significant differences between conditions (sample points in close spatial and temporal proximity), while effectively controlling for type I error. This statistical approach was used to evaluate the effects of direction (approach vs. avoidance) on the ERPs elicited by thing and person nouns. Accordingly, two separate cluster-based randomization tests were conducted.

**Figure 1:**
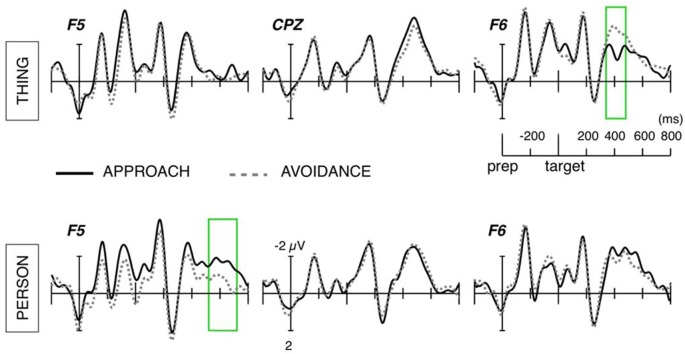
Averaged event-related potential (ERP) waveforms at electrodes representative of the anterior N400 and Frontal Negativity effects. Areas marked with green lines highlight the time window of the significant interaction between target and direction.

For thing nouns, the comparison between approach and avoidance directions yielded a significant cluster around the time window of the N400 component (350–470 ms after target noun onset; Tmaxsum = 904; *p* < 0.025), see Figure 1. This cluster reflected greater right frontal negative amplitudes for avoidance than for approach sentences. Further ANOVA showed, in this cluster, an interaction between direction and target, *F*_(22,1)_ = 14.05, *p* < 0.005, *η*^2^ = 0.39, which was apparently caused by the lack of significant effect of direction on person nouns. Thus, the effect in this right frontal N400 cluster seems to be specific to thing nouns (*p* < 0.001, *d* = 0.89).

For person-nouns, the comparison between approach and avoidance directions yielded a significant cluster around the time window of 545–750 ms (Tmaxsum = 462; *p* < 0.05), see Figure 1. This cluster reflects larger negative amplitudes at left frontal sites for person nouns preceded by approach verbs, relative to those preceded by avoidance verbs. The ANOVA on collapsed amplitude values revealed effects of direction, *F*_(22,1)_ = 10.06, *p* < 0.005, *η*^2^ = 0.33, and target, *F*_(22,1)_ = 5.1, *p* < 0.05, *η*^2^ = 0.19, with larger negative amplitudes by person-nouns than thing-nouns, and for nouns following approach verbs. The interaction did not reach significance, *F*_(22,1)_ = 2.95, *p* = 0.09, *η*^2^ = 0.12, however, follow-up comparisons confirmed that the effect of direction was specific to person-nouns (*p* < 0.01, *d* = 0.62).

### Source Estimation Analysis

We reanalyzed the ERP data within the 545–750 ms time window in order to estimate likely intracranial generators of the topographical difference between approach (person and thing sentences collapsed) and avoidance actions. We used the LAURA inverse solution approach (Local Auto-Regressive Average: Grave de Peralta Menendez et al., 2001). We averaged for each participant and condition the amplitude values within the selected time window. Then, these averaged values were submitted to distributed source analyses using LAURA (for a comparison of inverse solution methods, see Michel et al., 2004), implemented in Cartool software (Brunet et al., 2011). The solution space was calculated on a realistic head model that included 4,026 solution points, defined at regular distances within the gray matter of a standard MRI (Montreal Neurological Institute’s average brain). Current density magnitudes (ampere per square millimeter) at each solution point were calculated per subject and condition and submitted to statistical analyses using paired *t*-tests. Only *t*-test maps that showed differences below the statistical threshold of 0.005 for at least 15 nearby solution points were selected. For these statistically reliable *t*-test maps, regions of interest (ROI) were formed from the solution points showing the strongest differences and selected for further planned *t*-test comparisons and correlational analyses.

## Results

**Figure 2:**
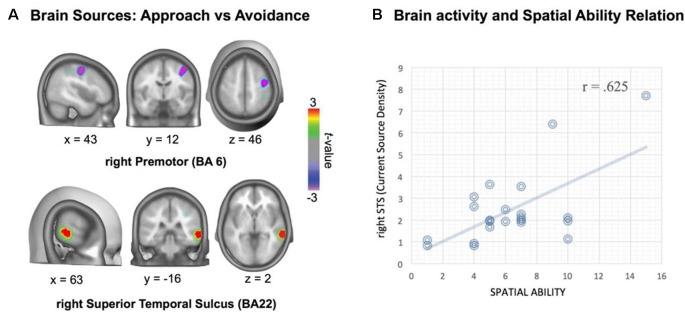
Source localization of approach-avoidance difference in the critical window ERP (545–750 ms), and association of individual differences in activations with spatial ability. Source localization indicates stronger activations for approach than avoidance at the anterior/middle right superior temporal sulcus (STS; BA21 and 22), and smaller activations for approach than avoidance at right middle frontal gyrus (BA6; **A**). Relation between spatial ability and temporal area activation: higher spatial ability is associated with stronger activation in approach action condition **(B)**.

There were two reliable brain sources showing differences between approach and avoidance actions: one at the right temporal lobe (Brodmann areas BA22 and BA21), overlapping the anterior/middle STS, with approach showing stronger activation than avoidance sentences, and the other at the right frontal medial gyrus (rFMG), overlapping part of the superior premotor area (SPA, BA6), in which approach showed less activation than avoidance sentences (see Figure 2A, Talairach coordinates of activation peaks).

Current density magnitudes were extracted in each ROI for each experimental condition: approach-person, approach-thing, avoidance-person and avoidance-thing. Planned comparisons showed greater activation in rSTS for approach-person condition than for avoidance-person condition (*Mdiff* = 0.58, *SD* = 1.21), *t*_(22)_ = 2.32, significant, *p* = 0.03, *d* = 0.48, whereas there was no significant approach-avoidance difference in the case of thing-target conditions, *p* > 0.20. Likewise, there was lesser activation in rFMG for approach-person condition than for avoidance-person condition (*Mdiff* = −0.56, *SD* = 1.01), *t*_(22)_ = 2.65, significantly, *p* = 0.015, *d* = 0.55, whereas there was no significant approach-avoidance difference in the case of thing-target conditions, *p* > 0.20. Thus, the difference activation of approach vs. avoidance found in these ROIs seems specifically associated with persons.

Current density magnitudes in each ROI for each experimental condition were taken for correlational analysis (see Cecchini et al., 2015 for a similar procedure; see also Berkman and Lieberman, 2010) with spatial ability. We found a moderate to high correlation (*r* = 0.625, *p* = 0.002) between rSTS activation in approach-person condition and spatial ability (Figure 2B). Regression analysis showed that only rSTS activation in approach-person condition correlated with spatial ability (*partial corr* = 0.625, *p* = 0.002), whereas the other conditions did not show significant partial correlations, *p* > 0.20. Likewise, there were no significant correlations for the source at the right premotor area with spatial ability, *p* > 0.20.

## Discussion

Our source estimation analysis showed two brain areas likely to be activated for processing approach vs. avoidance social relationship actions. In line with our expectations, the right STS was associated with greater activation for approach vs. avoidance. The STS is part of the mentalizing network that is recruited for processing action intentionality (Spunt et al., 2010; Dodell-Feder et al., 2011; Kennedy and Adolphs, 2012). In accordance with Iacoboni et al. (2004), activation of more anterior aspects of the STS could represent the process of giving a social-relational meaning to individual actions. In our study, likely activation of the STS would reflect brain processing of intentional approach to others during the reading of actions aimed at giving a relational meaning to them. By contrast, rMFG (BA6) was associated with less activation of approach vs. avoidance. As this area overlaps part of the SPA, we consider plausible that its activation could be associated with motor representations of approach and avoidance attitudes either pro stimulus (forward body movement) or against the stimulus (backward body movement), respectively (see Marrero et al., 2015).

The second aim of this study was to examine whether individual differences in brain encoding of approach and the ability for mental rotation of figures are associated. Our results support that rSTS activation, but not activation at rFMG, significantly correlated with spatial ability. As mentioned, the STS is recruited for processing action intentionality, and in our study, rSTS was associated with greater activation for approach vs. avoidance to persons. Thus, in accordance with our expectations, it would be the intentionality of approach that is associated with spatial ability. Plausibly, individual differences in brain encoding for intentional approach to others and spatial ability could be associated, as both processes would serve the same adaptive goal of secure interactions with others. This association could have an evolutionary explanation. In accordance with Barrett et al. (2010), different cognitive capacities likely coevolved for synergetic effects in enabling human cooperation and sociality. Thus, spatial ability and approach encoding could have coevolved for enabling human sociality inasmuch as individuals who both more deeply encode approach and move efficiently for self-protection in social encounters would increase their fitness and survival. In contrast, avoidance implies less interaction with avoided others. So, and in comparison to approach, its encoding is less relevant to the goal of having secure interactions, and thus significant correlation with spatial ability could be less expected.

Our correlational study is exploratory. Thus, further neuroscience research is necessary to examine the relationship between social approach and spatial ability.

## Author Contributions

HM was responsible for conception and design, and interpretation of data, drafting the article and revising it critically for important intellectual content. SY was responsible for the collection, analysis and interpretation of data. DB was responsible for design, analysis and interpretation of data, and revising the article critically for important intellectual content. EG and JD were responsible for main conception and design, analysis and interpretation of data, drafting the article and revising it critically for important intellectual content. MU was responsible for conception and design, interpretation of data, and revising the article critically for important intellectual content.

## Conflict of Interest Statement

The authors declare that the research was conducted in the absence of any commercial or financial relationships that could be construed as a potential conflict of interest.
